# Association of Trimethylamine N-Oxide and Its Precursor With Cerebral Small Vessel Imaging Markers

**DOI:** 10.3389/fneur.2021.648702

**Published:** 2021-04-01

**Authors:** Yiyi Chen, Jie Xu, Yuesong Pan, Hongyi Yan, Jing Jing, Yingying Yang, Xing Wang, Huijuan Wan, Ying Gao, Shangrong Han, Xi Zhong, Chenhui Liu, Jingtao Pi, Zhengyang Li, Biyang Luo, Guangyao Wang, Yilong Zhao, Nan Wang, Jinxi Lin, Xia Meng, Xingquan Zhao, Liping Liu, Wei Li, Yong Jiang, Zixiao Li, Xinmiao Zhang, Xiaomeng Yang, Ruijun Ji, Chunjuan Wang, Hao Li, Penglian Wang, Huaguang Zheng, Weizhong Ji, Xueli Cai, Songdi Wu, Xinsheng Han, Yongjun Wang, Yilong Wang

**Affiliations:** ^1^Department of Neurology, Beijing Tiantan Hospital, Capital Medical University, Beijing, China; ^2^China National Clinical Research Center for Neurological Diseases, Beijing, China; ^3^Center of Stroke, Beijing Institute for Brain Disorders, Beijing, China; ^4^Beijing Key Laboratory of Translational Medicine for Cerebrovascular Disease, Beijing, China; ^5^Tiantan Neuroimaging Center of Excellence, Beijing, China; ^6^Department of Neurology, Qinghai Province People's Hospital, Qinghai, China; ^7^Department of Neurology, Lishui Central Hospital, Lishui, China; ^8^Department of Neurology, The First Hospital of Xi'an, Xi'an, China; ^9^Department of Neurology, Kaifeng Central Hospital, Kaifeng, China

**Keywords:** trimethylamine N-oxide, choline, cerebral small vessel disease, white matter hyperintensities, cerebral microbleeds, lacunes

## Abstract

**Background:** High plasma levels of trimethylamine N-oxide (TMAO) and its precursor choline have been linked to stroke; however, their association with cerebral small vessel disease remains unclear. Here we evaluated the association of plasma levels of TMAO and choline with imaging markers of cerebral small vessel disease, including white matter hyperintensities, lacunes, and cerebral microbleeds.

**Methods:** We performed a baseline cross-sectional analysis of a multicenter hospital-based cohort study from 2015 to 2018. The data were collected from 30 hospitals in China and included 1,098 patients with ischemic stroke/transient ischemic attack aged ≥18 years. White matter hyperintensities, lacunes, and cerebral microbleeds were evaluated with the patients' demographic, clinical, and laboratory information removed. White matter hyperintensities were rated using the Fazekas visual grading scale, while the degree of severity of the lacunes and cerebral microbleeds was defined by the number of lesions.

**Results:** Increased TMAO levels were associated with severe white matter hyperintensities [adjusted odds ratio (aOR) for the highest vs. lowest quartile, 1.5; 95% confidence interval (CI), 1.0–2.1, *p* = 0.04]. High TMAO levels were more strongly associated with severe periventricular white matter hyperintensities (aOR for the highest vs. lowest quartile, 1.6; 95% CI, 1.1–2.3, *p* = 0.009) than deep white matter hyperintensities (aOR for the highest vs. lowest quartile, 1.3; 95% CI, 0.9–1.9, *p* = 0.16). No significant association was observed between TMAO and lacunes or cerebral microbleeds. Choline showed trends similar to that of TMAO in the association with cerebral small vessel disease.

**Conclusions:** In patients with ischemic stroke or transient ischemic attack, TMAO and choline appear to be associated with white matter hyperintensities, but not with lacunes or cerebral microbleeds; TMAO and choline were associated with increased risk of a greater periventricular, rather than deep, white matter hyperintensities burden.

## Introduction

Cerebral small vessel disease (CSVD) refers to a spectrum of imaging changes affecting the brain's small vessel network. The presence of CSVD increases with age and contributes to approximately 45% of dementia and 20% of stroke cases, which affects the late-life quality of the aging population (1–4). To date, the pathophysiology of CSVD remains debatable. Although studies have shown that traditional risk factors for stroke are also associated with the prevalence of CSVD, treatments with anti-hypertensive agents or statins in CSVD patients have not shown efficacy (5–8).

Gastrointestinal tract microbiota form a complex ecosystem and modulate the homeostatic metabolic balance (9). Trimethylamine N-oxide (TMAO) is a gut microbial-derived metabolite and has been found in the brain, indicating that it has the ability to pass through the blood-brain barrier (BBB) (10). Previous studies have suggested that TMAO increases platelet hyperactivity, oxidative stress, and endothelial inflammatory responses; on the other hand, it decreases nitric oxide production and downregulates inter-endothelial tight junction proteins, ultimately disrupting BBB integrity (11–13).

We hypothesized that following increased levels of circulating TMAO and its precursor, choline, these metabolites could interact with endothelial cells within the BBB, leading to cellular injury and influencing the intracellular expression of tight junction proteins. These combined effects may disrupt the BBB and eventually promote the formation of white matter hyperintensities (WMHs), lacunes, or cerebral microbleeds (CMBs). Based on this hypothesis, we have conducted a cross-sectional study to evaluate the association of plasma TMAO levels and its precursor, choline, with the imaging markers of CSVD, including WMHs, lacunes, and CMBs.

## Methods

### Study Design and Participants

In China, 30 hospitals were invited to participate in this study between August 2015 and March 2018. The geographical distribution of the study sites is illustrated in Supplementary Figure 1. A total of 1,159 patients, aged ≥ 18 years, and within 7 days of onset of ischemic stroke (IS) or transient ischemic attack (TIA), who provided consent for CSVD imaging evaluation and blood sample collection, were consecutively enrolled. Patients with measurable neurologic deficits were diagnosed with acute IS based on World Health Organization criteria and confirmed brain imaging [computerized tomography or magnetic resonance imaging (MRI)] (14). Sixty-one patients with missing TMAO and choline data or unqualified MRI scans were excluded, resulting in a total of 1,098 patients to be included in the final analysis. The study was approved by the Ethics Committee at Beijing Tiantan Hospital. All patients provided written informed consent before enrolment in this study.

### TMAO and Choline Measurement

Fasting blood samples were collected within 24 h of enrollment and centrifuged into serum, plasma, and white blood cells in a local laboratory. These extracted samples were transported and stored in a −80°C freezer in Beijing Tiantan Hospital until analysis. Plasma TMAO and choline concentrations were determined using liquid chromatography-mass spectrometry on QTRAP 5500 (AB Sciex Pte. Ltd, Framingham, MA, USA) using an internal standard of d9-TMAO and d9-choline in methanol. The detailed methodology applied is available in Supplementary Material. Laboratory test results were uploaded into an electronic capture system (EDC), and all data were de-identified before data analysis.

### Clinical Assessment

For each patient, we recorded information on traditional risk factors for both stroke and CSVD including age; sex; body mass index (BMI); systolic and diastolic blood pressure (SBP and DBP, respectively); medication history of anti-platelet agents, lipid-lowering agents, and anti-hypertensive agents; prior diagnosis of hypertension; diabetes mellitus (DM); stroke or TIA; and smoking. Furthermore, we recorded each patient's laboratory data, including estimated glomerular filtration rate [eGFR, calculated as 186 × (serum creatinine)^−1.154^ × (age)^−0.203^ (× 0.742 if women)] and levels of high-density lipoprotein cholesterol (HDL-C), low-density lipoprotein cholesterol (LDL-C), high-sensitivity C-reactive protein (hs-CRP), and homocysteine (Hcy).

Hypertension was defined as an SBP ≥ 140 mm Hg and/or DBP ≥ 90 mm Hg, on at least two separate occasions, or the current use of anti-hypertensive medications. DM was defined as a fasting plasma glucose level ≥ 126 mg/dl (7.0 mmol/L) and/or current use of hypoglycemic agents. The calculation of BMI was based on the patient's weight and height (kg/m^2^). Smoking status was defined by current smoking.

### Imaging Assessment

CSVD neuroimaging markers of interest in this study were WMHs, lacunes, and CMBs. The operators who rated the images were blinded to all patients' demographic, clinical, and laboratory data. Radiological assessment of each CSVD imaging marker was performed by two experienced neurologists, following the imaging protocol, and independently documented in the EDC. If an acute infarct lesion influenced the operator's decision regarding the CSVD assessment, they would select the “unable to determine” option on the EDC and an independent statistician would then exclude these data from our analysis. After screening all images, inconsistent results were assessed by a senior neuro-radiologist who was blinded to the initial results. The imaging data were saved in Digital Imaging and Communications in Medicine or DICOM format and evaluated by utilizing a RadiANT DICOM Viewer (Medixant Ltd, Poznan, Poland). WMHs, lacunes, and CMBs were defined according to the STandards for ReportIng Vascular changes on nEuroimaging or STRIVE criteria (3). Brain MRI was performed on either a 1.5 Tesla or 3.0 Tesla MRI scanner, as available. Detailed MRI acquisition included (1) T1-weighted images, (2) T2-weighted images, (3) T2 fluid-attenuation inversion recovery (FLAIR) images, and (4) susceptibility-weighted imaging (SWI), or T2^*^-weighted gradient-recall echo images if SWI was not provided.

WMHs of presumed vascular origin were defined as patchy areas with hyperintensities on T2-weighted and FLAIR sequences and hypointensities on T1-weighted images. T2-weighted and FLAIR sequences were analyzed using Fazekas semiquantitative visual grading scale. This method assesses periventricular (contiguous with the ventricle margin) and deep (not contiguous with the ventricles) white matter changes separately, according to the following stages: 0 (absence), 1 (pencil-thin lining and non-confluent), 2 (confluent), and 3 (diffuse). The sum of the periventricular and deep WMHs scores was used to characterize patients' WMH burden level, and were classified as “None” (total Fazekas score of 0), “Low” (1, 2), “Medium” (3, 4), and “High” (5, 6).

The number of lacunes and CMBs was coded as “None” (0 lacunes/CMBs), “A few” (1–2 lacunes/CMBs), and “Many” (3 or more lacunes or CMBs). The presence of lacunes and CMBs was evaluated in both hemispheres; lacunes were assessed in the subcortical white matter, basal ganglia, thalamus, and infratentorial areas (the pons), and CMBs were additionally assessed in the lobar and cerebellum regions. Lacunes are asymptomatic and well-defined lesions of 3–15 mm. These fluid-filled cavities commonly have the same signal as cerebrospinal fluid—they are hyperintense on T2-weighted images and hypointense on T1-weighted and FLAIR sequences, with an occasional hyperintense rim. CMBs appear as focal hemosiderin deposits caused by blood leakage from damaged arteriolar walls. They are defined as <10 mm-size ovoid or round lesions with a hypointense signal on T2^*^-weighted gradient sequences or SWI. CMB mimics, such as vessels or calcifications, were excluded from our analysis.

### Statistical Analyses

Continuous variables are expressed as the median (interquartile range), and categorical variables as numbers (percentages). Differences in the distribution of categorical variables and non-normally distributed data were tested using a χ2 test and Kruskal–Wallis test, respectively. The Student's *t*-test was used to compare normally distributed variables between groups. Ordinal logistic regression analysis was performed to determine the association of plasma TMAO and choline levels with the ordinal CSVD imaging markers (i.e., the four WMHs Fazekas rating levels or number of lesions). The regression models were adjusted for conventional vascular risk covariates and those associated with the severity of CSVD, including age; sex; history of hypertension; DM; prior stroke or TIA; history of anti-platelet, lipid-lowering, or anti-hypertensive agent usage; BMI; SBP; LDL-C; eGFR; and inflammatory markers such as hs-CRP and Hcy. Patients with missing covariate data were excluded from the analyses. Statistical analyses were performed with SPSS version 25.0 (IBM, Armonk, NY, USA) and SAS 9.4 software (SAS Institute, Cary, NC, USA) by independent statisticians. A *p*-value < 0.05 was considered significant.

## Results

### Patient Clinical Characteristics

Our study included 1,098 patients with IS or TIA [mean age 62 (54, 69) years, men 71%]. Among all patients, 64% (*n* = 704) had a prior diagnosis of hypertension and 26% (*n* = 284) had a previous stroke or TIA. Accordingly, previous use of anti-hypertensive agents was identified in 45% (*n* = 498) of patients, while 18% (*n* = 195) had used anti-platelet agents and 11% (*n* = 125) had used lipid-lowering agents. The median levels of TMAO and choline were 1.7 (1.2–2.5) μmol/L and 13.5 (11.4–16.2) μmol/L, respectively. Plasma TMAO levels did not differ between stroke subtypes (Supplementary Table 1). Table 1 displays patients' baseline characteristics.

**Table 1:** Characteristics of the study population.

**Parameters**	**All patients (*n* = 1,098)**
**Demographics**	
Age, years	62 (54, 69)
Male sex	774 (70.5)
**Vascular risk factors**	
BMI, kg/m^2^	24.7 (22.9, 26.7)
Systolic BP, mmHg	148 (133, 164)
Diastolic BP, mmHg	88 (79, 95)
Current smoker	378 (34.4)
Hypertension	704 (64.1)
Diabetes mellitus	254 (23.1)
Prior Stroke/TIA	284 (25.9)
**Medical use history**	
Anti-platelet agent	195 (17.8)
Lipid-lowering agent	125 (11.4)
Anti-hypertensive agent	498 (45.4)
**Laboratory tests**	
eGFR, mL/min/1.73 m^2^	94.6 (83.7, 102.6)
LDL-C, mmol/L	2.2 (1.6, 3.0)
HDL-C, mmol/L	0.9 (0.8, 1.1)
hs-CRP, mg/L	1.5 (0.7, 3.8)
Hcy, μmol/L	16.8 (13.3, 23.1)
TMAO, μmol/L	1.7 (1.2, 2.5)
Choline, μmol/L	13.5 (11.4, 16.2)

*Data are presented as median (interquartile range) or number (percentages)*.

### Plasma TMAO and Choline Identified as Risk Factors for WMHs

Based on their Fazekas visual rating scores, patients classified as having “No” or a “Low,” “Medium,” or “High” WMH burden comprised 6% (*n* = 67), 52% (*n* = 570), 24% (*n* = 262), and 18% (*n* = 199) of all patients, respectively. The total WMH burden was significantly higher among patients with older age, hypertension, prior stroke or TIA, history of anti-platelet or anti-hypertensive agent usage, lower eGFR levels, and higher SBP, hs-CRP, Hcy, TMAO, and choline levels (Supplementary Table 2).

**Figure 1:**
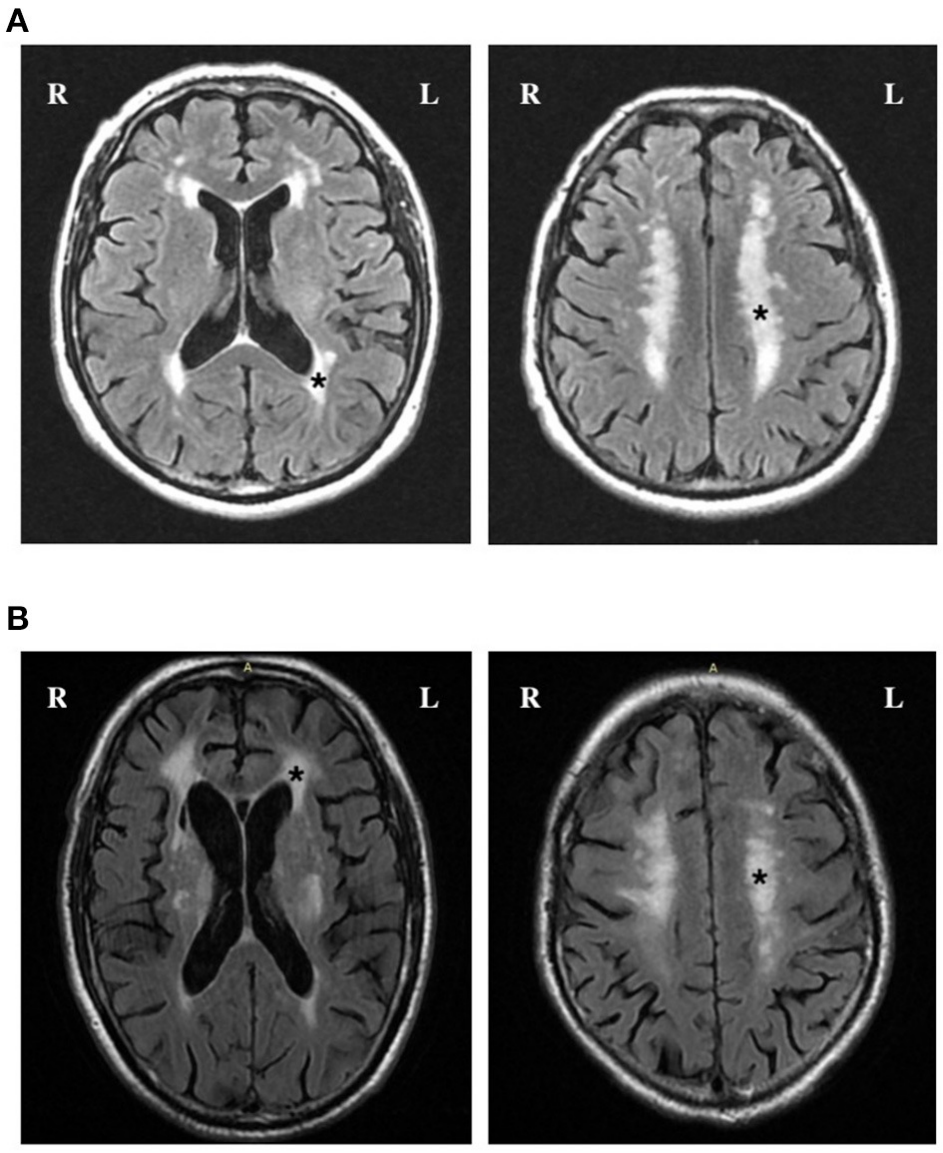
FLAIR images of high total WMH burden in patients with elevated TMAO levels. **(A)** and **(B)** Axial FLAIR slices show the position of periventricular (left) and deep WMH (right). Fazekas scores in periventricular region and deep region were both rated as grade 3 (^*^) in a patient with 5.3 μmol/L TMAO **(A)** and 5.4 μmol/L TMAO **(B)**.

Figure 1 shows FLAIR images of “High” total WMH burden in patients with elevated TMAO levels. When TMAO and choline were assessed as quartiles, compared with the lowest quartile, the adjusted odds of a higher total WMH burden were greater in patients with levels of both circulating TMAO > 2.5 μmol/L and choline > 16.2 μmol/L (Table 2). We further characterized the associations of periventricular and deep WMHs with TMAO and choline separately. Compared with the subjects in the lowest quartile, we found that patients with TMAO > 2.5 μmol/L or choline levels > 16.2 μmol/L displayed an increased risk of having greater periventricular WMH burden-−1.6-fold [95% confidence interval (CI), 1.1–2.3] and 1.4-fold (95%CI, 1.0–2.0), respectively. However, no significant association was found between patients with either TMAO > 2.5 μmol/L [odds ratio (OR) = 1.3; 95% CI, 0.9–1.9] or choline levels > 16.2 μmol/L (OR = 1.4; 95% CI, 1.0–2.0) and the risk of having a greater deep WMH burden (Table 2).

**Table 2:** Relationships between plasma TMAO and choline concentrations and the risks of higher total, periventricular, and deep WMH burden.

	**Total WMH Burden**		**Periventricular WMH Burden**		**Deep WMH Burden**	
	**Unadjusted^a^ OR (95% CI)**	**Adjusted^b^ OR (95% CI)**	**Unadjusted^a^ OR (95% CI)**	**Adjusted^b^ OR (95% CI)**	**Unadjusted^a^ OR (95% CI)**	**Adjusted^b^ OR (95% CI)**
**TMAO (range**, ***μ*****mol/L)**						
1st quartile (<1.2)	1.0 (ref)	1.0 (ref)	1.0 (ref)	1.0 (ref)	1.0 (ref)	1.0 (ref)
2nd quartile (1.2, 1.7)	1.3 (0.9, 1.8)	0.9 (0.6, 1.3)	1.4 (1.0, 1.9)	1.0 (0.7, 1.4)	1.2 (0.9, 1.7)	0.9 (0.6, 1.3)
3rd quartile (1.7, 2.5)	1.6 (1.2, 2.2)	1.3 (0.9, 1.8)	1.7 (1.2, 2.3)	1.3 (0.9, 1.8)	1.4 (1.0, 2.0)	1.2 (0.8, 1.7)
4th quartile (>2.5)	2.4 (1.8, 3.4)	1.5 (1.0, 2.1)	2.7 (1.9, 3.7)	1.6 (1.1, 2.3)	2.1 (1.5, 2.9)	1.3 (0.9, 1.9)
**Choline (range**, ***μ*****mol/L)**						
1st quartile (<11.4)	1.0 (ref)	1.0 (ref)	1.0 (ref)	1.0 (ref)	1.0 (ref)	1.0 (ref)
2nd quartile (11.4, 13.5)	1.2 (0.9, 1.7)	1.0 (0.7, 1.4)	1.2 (0.9, 1.7)	1.0 (0.7, 1.4)	1.3 (0.9, 1.8)	1.0 (0.7, 1.5)
3rd quartile (13.5, 16.2)	1.4 (1.0, 1.9)	1.2 (0.8, 1.7)	1.4 (1.0, 2.0)	1.2 (0.8, 1.7)	1.3 (1.0, 1.9)	1.1 (0.8, 1.6)
4th quartile (>16.2)	2.0 (1.5, 2.8)	1.5 (1.0, 2.1)	2.0 (1.5, 2.7)	1.4 (1.0, 2.0)	1.9 (1.3, 2.6)	1.4 (1.0, 2.0)

a *Ordinal logistic regression*.

b *Model adjusted for age, sex, hypertension, diabetes mellitus, prior stroke or transient ischemic attack, history of anti-platelet, lipid-lowering, or anti-hypertensive agents, BMI (body mass index), systolic blood pressure, low-density lipoprotein cholesterol, estimated glomerular filtration rate, homocysteine, and high sensitive-C-reactive protein. TMAO, trimethylamine N-oxide; OR, odds ratio; CI, confidence interval*.

**Figure 2:**
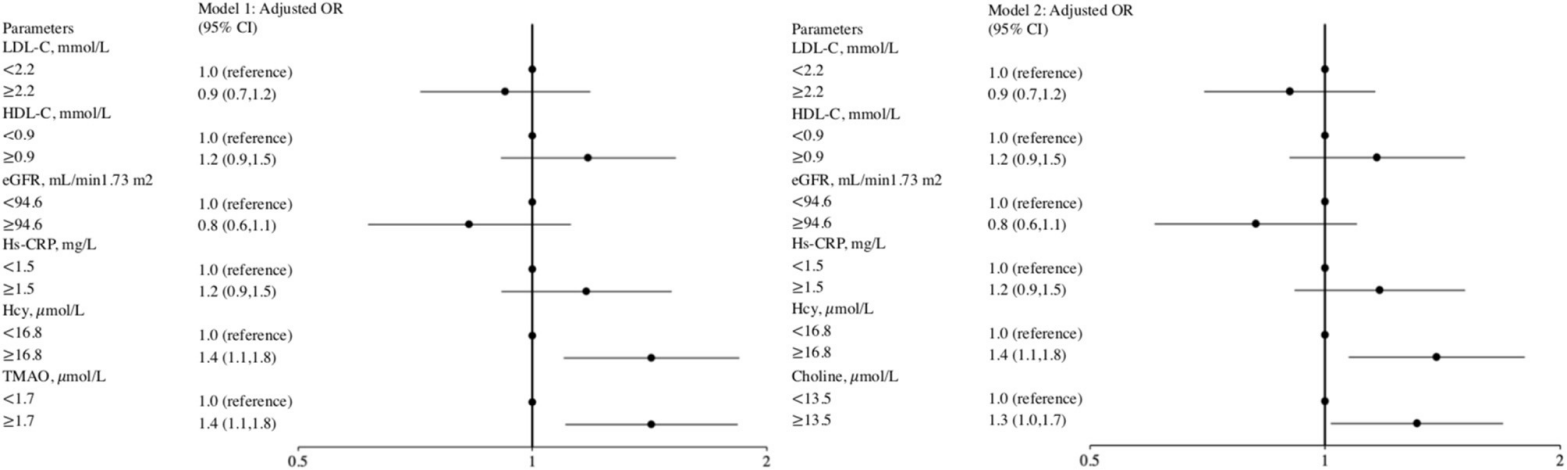
Forest plot of the ORs for potential biomarkers with severe total WMH risk. The odds ratio was calculated according to the median value of potential biomarkers' levels using ordinal logistic regression models. The bar represents 95% confidence interval (CI). Model 1 included age, sex, hypertension, diabetes mellitus, prior stroke or transient ischemic attack, history of anti-platelet, lipid-lowering, or anti-hypertensive agents, BMI (body mass index), systolic blood pressure, low-density lipoprotein cholesterol, estimated glomerular filtration rate, homocysteine, and high sensitive-C-reactive protein and TMAO as covariates. Model 2 included age, sex, hypertension, diabetes mellitus, prior stroke or transient ischemic attack, history of anti-platelet, lipid-lowering, or anti-hypertensive agents, BMI, systolic blood pressure, low-density lipoprotein cholesterol, estimated glomerular filtration rate, homocysteine, and high sensitive-C-reactive protein and choline as covariates.

Ordinal logistic regression analysis found that elevated levels of the inflammation marker Hcy, but not of hs-CRP or other potential biomarkers, were associated with a greater total WMH burden (Figure 2). We evaluated whether a combination of Hcy and either TMAO or choline levels could be used as a predictor for WMHs. Patients were categorized into four groups according to median TMAO and Hcy values. This analysis showed that subjects with both elevated TMAO and Hcy levels displayed a significant 2.0-fold (95% CI, 1.4–3.0) increase in the risk of presence of a greater total WMH burden, compared with patients with low levels of both TMAO and Hcy (reference group) (Table 3). Moreover, high TMAO levels were associated with significantly increased risk of higher Fazekas scores for total WMHs (1.7-fold risk for total WMHs when TMAO levels were high; 95% CI, 1.2–2.4) among patients with low Hcy levels. Similarly, high Hcy levels were associated with significantly increased risk of higher Fazekas scores for total WMHs (1.7-fold risk for total WMHs when Hcy levels were high, 95% CI, 1.2–2.4) among patients with low TMAO levels. Likewise, subjects with both high choline and Hcy levels displayed a significant 1.8-fold (95% CI, 1.3–2.6) increased risk of presence of greater total WMH burden, compared with patients with low levels of both choline and Hcy (reference group). Among patients with low Hcy levels, the association between high choline levels and higher Fazekas scores for total WMHs were marginally significant (1.4-fold risk for total WMHs when choline levels were high; 95% CI, 1.0–2.0). Among patients with low choline levels, high Hcy levels were associated with a significantly increased risk of higher Fazekas scores for total WMHs (1.5-fold risk for total WMH when Hcy levels were high; 95% CI, 1.0–2.1) (Table 3).

**Table 3:** Relationships between plasma TMAO or choline concentrations, plasma Hcy levels, and the risk of a higher total WMH burden in the context of plasma Hcy levels.

		**Total WMH Burden**	
		**Unadjusted^a^ OR (95% CI)**	**Adjusted^b^ OR (95% CI)**
**TMAO**	**Hcy**		
Low	Low	1.0 (ref)	1.0 (ref)
Low	High	1.9 (1.4, 2.6)	1.7 (1.2, 2.4)
High	Low	2.0 (1.4, 2.8)	1.7 (1.2, 2.4)
High	High	2.8 (2.0, 3.8)	2.0 (1.4, 3.0)
**Choline**	**Hcy**		
Low	Low	1.0 (ref)	1.0 (ref)
Low	High	1.7 (1.2, 2.4)	1.5 (1.0, 2.1)
High	Low	1.6 (1.1, 2.2)	1.4 (1.0, 2.0)
High	High	2.3 (1.7, 3.1)	1.8 (1.3, 2.6)

*Patients were categorized into four groups according to median TMAO and Hcy values*.

a *Ordinal logistic regression*.

b *Model adjusted for age, sex, hypertension, diabetes mellitus, prior stroke or transient ischemic attack, history of anti-platelet, lipid-lowering, or anti-hypertensive agents, BMI (body mass index), systolic blood pressure, low-density lipoprotein cholesterol, and estimated glomerular filtration rate. TMAO, trimethylamine N-oxide; OR, odds ratio; CI, confidence interval*.

### The Association of Plasma TMAO and Choline Levels With Lacunes

Lacunes were present in 54% (*n* = 592) of patients: 28% (*n* = 304) had one or two lacunes, and 26% (*n* = 288) had three or more. Patients who had a greater number of lacunes were more advanced in age; had higher frequencies of current smoking status; hypertension; prior stroke or TIA; medical use history of anti-platelet agents, lipid-lowering agents, or anti-hypertensive agents; lower eGFR levels; and higher levels of BMI, Hcy, TMAO, and choline (Supplementary Table 3). Compared with patients with TMAO levels <1.2 μmol/L, patients with TMAO levels >2.5 μmol/L had a 1.7-fold increased risk of a greater number of lacunes (95% CI, 1.2–2.3). However, adjusting for traditional risk factors greatly attenuated the association. Consistently, compared with subjects with choline levels <11.4 μmol/L, patients with levels >16.2 μmol/L showed a 1.6-fold increase in the odds of greater numbers of lacunes (95% CI, 1.1–2.1). However, further addition of traditional risk factors to the model neutralized the association (Supplementary Table 4).

### The Association of Plasma TMAO and Choline Levels With CMBs

CMBs were found in 32% (*n* = 349) of the patients: 16% (*n* = 181) had one or two CMBs and 15% (*n* = 168) had three or more CMBs. Patients with higher numbers of CMBs were more advanced in age and were more likely to have a history of hypertension; DM; prior stroke or TIA; medical use of either anti-platelet agents, lipid-lowering agents, or anti-hypertensive agents; and displayed higher levels of SBP, DBP, and Hcy (Supplementary Table 3). In our study, neither TMAO nor choline levels increased the odds of a higher number of CMBs (Supplementary Table 5).

## Discussion

Our study reports an association between circulatory TMAO and choline levels and CSVD, encompassing WMHs, lacunes, and CMBs. We demonstrated that: (1) elevated plasma levels of TMAO and choline are each associated with a higher risk of WMHs, independent of traditional risk factors, and are each more closely associated with periventricular WMHs than deep WMHs; and (2) plasma TMAO and choline levels in our study appeared to have no significant association with the presence of lacunes or CMBs after adjusting for confounding variables.

Evidence for an association between microbiota metabolites and CSVD is limited and conflicting. The Framingham offspring cohort showed that estimated dietary choline intake from a food-frequency questionnaire was inversely associated with WMH volume (15). However, a community-based study conducted in Boston observed a non-significant association between plasma choline levels and WMH volume (16). Our results suggest that the levels of plasma TMAO and its precursor, choline, in IS/TIA patients remain significantly associated with WMHs, particularly in the periventricular white matter, after adjusting for both vascular risk factors, eGFR and inflammatory factors. We speculate that TMAO or choline participates in endothelial dysfunction, inducing or reinforcing the inflammatory cascade and disrupting the BBB integrity. On one hand, after adjustment for traditional vascular risk factors, elevated levels of circulating TMAO have been recognized as an indicator of endothelial dysfunction. TMAO supplementation increases oxidative stress and activates the p53/p21/Rb pathway which mediates cellular senescence, thereby resulting in reduced nitric oxide bioavailability (17–19). On the other hand, TMAO triggers nucleotide-binding oligomerization domain–like receptor family pyrin domain–containing 3 (NLRP3) inflammasome formation, further exacerbating endothelial injuries with subsequent inflammatory cytokines production (20, 21). Meanwhile, TMAO induces the activation of NLRP3 inflammasomes, which is mediated by the release of high-mobility group box 1, which in turn activates toll-like receptor 4 (TLR4), and subsequently downregulates inter-endothelia tight junction proteins such as ZO-2, occludin and VE-cadherin *in vitro* (13). BBB disruption in normal appearing WM predicts future white matter lesion (22). The permeability of the BBB is closely associated with each of its constituent elements, especially the most critical player, endothelial cells. Notably, paracellular tight junction complexes in line with endothelial cells are also vital to the integrity and function of the BBB (10, 23, 24). A study using an animal model of CSVD has reported that the destruction of tight junction proteins is closely associated with the severity of WMHs (25). Taken together, the abovementioned mechanisms might possible explain our clinical observations. Additionally, our study found that IS/TIA patients with both high TMAO (choline) and high Hcy levels had the highest proportion of severe WMHs. Red meat, fish, nuts, yolk, and eggs are rich in dietary choline. Certain gut bacteria metabolize trimethylammonium-containing nutrients into trimethylamine, which is ultimately oxidized to TMAO (26–29). Different dietary patterns have different impacts on the plasma levels of TMAO, choline, and Hcy (30–32). Previous studies have suggested that plasma Hcy concentration was strongly associated with WMHs, and Hcy-lowering therapy significantly slowed WMHs progression (33–35). Therefore, dietary interventions targeting plasma TMAO, choline, and Hcy levels, and their impact on CSVD should be investigated in the future.

Our data observed no significant associations of TMAO or choline levels with the numbers of lacunes or CMBs. High circulating TMAO levels induce platelet hyperresponsiveness and increase the risk of thrombotic events. Higher coagulability may explain the attenuated association between elevated TMAO and choline levels and the number of CMBs (11, 36). In this study, patients were allocated according to the number of CMBs instead of by CMBs regions. CMBs on lobar or deep locations are associated with differed pathogenesis, mainly cerebral amyloid angiopathy and hypertensive arteriopathy, respectively (37). Since vascular inflammation primarily participates in the hypertensive arteriopathy in brain regions such as the basal ganglia, future studies should further analyze the association of TMAO and choline with the region-specific of CMBs distribution (38). Higher plasma choline levels have been shown to be associated with a lower incidence of small vessel subcortical infarcts (16). According to the Trial of Org 10172 in Acute Stroke Treatment or TOAST criteria, plasma TMAO levels did not differ significantly between stroke subtypes, and no definite association has been reported between TMAO and small vessel occlusion (39–42). Accordingly, the lack of obvious associations between TMAO levels and lacunar infarcts may partially explain the non-significant association between plasma TMAO or choline levels with the number of lacunes that we found in our study. Endothelia injuries play a central role in BBB function. It would induce adherent activation of coagulatory state which is also consistent with finding of restricted thrombus formation and constant plasma enter the vessel wall, and ultimately angiopathy with small vessel occlusion in brain (43). However, only one fifth of acute lacunar infarcts progressed to lacunes after stroke onset on a median follow-up imaging of <1 year. Although over 90% of lacunes are found adjacent to WMHs (44–46), lacunes formation are complex and not complete understood. Meanwhile, it has been suggested that lacunes represent as an extreme end of small vessel changes (47, 48). Given the inconsistent finding of TMAO with WMHs and lacunes, pathological processes underlies WMHs and lacunes may not be similar.

Our study has some limitations. First, due to its cross-sectional nature, we could not examine the development or possible causal association between TMAO and choline levels and CSVD. We expect that future longitudinal studies and Mendelian randomized studies will validate our hypothesis. Second, we did not collect fecal samples to determine microbiota composition. Nonetheless, the association between microbiota and TMAO has been investigated extensively (49). Third, we were unable to directly evaluate the impact of diet on TMAO, choline, and Hcy as information on dietary habits was not collected in this study. Finally, our samples were obtained from stroke patients, thereby limiting the generalizability of our results to the general population. However, as the data were obtained from 30 hospitals, we feel that they are a valid general representation of patients with stroke.

## Conclusions

Our results suggests that in IS/TIA patients, TMAO and its precursor choline appear to be associated with WMHs, but not with lacunes and CMBs. Elevated TMAO and choline levels particularly seem to increase the risk of having greater periventricular WMH burden more than deep WMH burden. Thus, gut microbial-derived metabolites appear to have an effect on WMHs. Confirmation of these findings through further longitudinal research or dietary interventions on patients with CSVD might have important implications for clinical practice.

## Data Availability Statement

The original contributions presented in the study are included in the article/Supplementary Material, further inquiries can be directed to the corresponding author/s.

## Ethics Statement

The studies involving human participants were reviewed and approved by the study was approved by the Ethics Committee at Beijing Tiantan Hospital. The patients/participants provided their written informed consent to participate in this study.

## Author Contributions

YiW had full access to all of the data in the study and takes responsibility for the integrity of the data and the accuracy of the data analysis. YC and YiW: concept and design. YC, JX, JJ, YY, XW, HW, YG, SH, XZ, CL, JP, ZL, BL, GW, YZ, NW, WJ, XC, SW, and XH: acquisition, analysis, or interpretation of data. YC: drafting of the manuscript. JX and YiW: critical revision of the manuscript for important intellectual content. YP and HY: statistical analysis. YoW, JX, HZ, PW, and YiW: obtained funding. JL, XM, XZ, LL, WL, YJ, ZL, XZ, XY, RJ, CW, and HL: administrative, technical, or material support. YiW: supervision. All authors contributed to the article and approved the submitted version.

## Conflict of Interest

The authors declare that the research was conducted in the absence of any commercial or financial relationships that could be construed as a potential conflict of interest.
