# Supplementary material for: Association of Trimethylamine N-Oxide and Its Precursor With Cerebral Small Vessel Imaging Markers
Source: Front Neurol. 2021 Apr 1;12:648702. doi: 10.3389/fneur.2021.648702 (PMC8047127; doi:10.3389/fneur.2021.648702)
Supplement: Supplementary file 1 [file Data_Sheet_1.docx]

Supplementary Material

1. Expanded Methods of trimethylamine-N-Oxide (TMAO) LC/MS/MS measurement
2. Supplementary Figure 1 Geographical locations of study sites.
3. Supplementary Table 1 Plasma TMAO levels between stroke subtypes
4. Supplementary Table 2 Demographics and baseline characteristics of patients divided into four groups based on the severity of WMH according to the Fazekas scale
5. Supplementary Table 3 Comparison of demographics and baseline characteristics of patients divided into three groups based on the number of lacunes or CMBs.
6. Supplementary Table 4 Relationships between plasma TMAO and choline concentrations and risk of having greater number of lacunes
7. Supplementary Table 5 Relationships between plasma TMAO and choline concentrations and risk of having greater number of CMBs

**Supplementary Data**

**Expanded Methods of trimethylamine-N-Oxide (TMAO) LC/MS/MS measurement**

The plasma levels of trimethylamine N-oxide (TMAO) were measured as follows: first, 20 μL of plasma were mixed with 80 μL of a 5-μM internal standard comprised of d9-TMAO in methanol. Proteins were precipitated, and the supernatant was recovered following centrifugation at 20,000 g at 4°C for 10 min. Supernatants (70 μL) were analyzed by injection onto a silica column (2.0 × 150 mm, Luna 5u Silica 100 A; Cat. No. 00F-4274-B0, Phenomenex, Torrance, CA, USA) at a flow rate of 0.4 mL/min using an LC-20AD Shimadzu pump system, SIL-20AXR autosampler interfaced with an API 5500Q-TRAP mass spectrometer (AB SCIEX, Framingham, MA, USA). A discontinuous gradient was generated to resolve the analytes by mixing solvent A (0.1% formic acid and 10 mM ammonium format in water) with solvent B (0.1% formic acid in acetonitrile). Analytes were monitored using electrospray ionization in positive-ion mode with multiple reaction monitoring of precursor and characteristic product-ion transitions of TMAO at m/z 76→58 and d9-TMAO at m/z 85→66, respectively. Standard curves were deemed acceptable if the coefficient of determination (R2) was 0.999. Accuracy percentages are displayed in Table I. Quality control was measured every 20 samples. The calculated means and coefficient of variation (CV%) are listed in Table II.

**Table I** Accuracy (%) of TMAO determination by liquid chromatography-mass spectrometry/MS

| **Standard concentration (*µ*mol/L)** | **Accuracy of TMAO (%)** |
| --- | --- |
| 0.195 | 91 |
| 0.39 | 91.3 |
| 0.78 | 90 |
| 1.56 | 96.9 |
| 3.13 | 104 |
| 6.25 | 101 |
| 12.5 | 99.5 |

TMAO = trimethylamine N-oxide.

**Table II** Coefficient of variation (CV%) of TMAO quality controls (QC)

|  | | **QC1** | **QC2** | **QC3** |
| --- | --- | --- | --- | --- |
| TMAO | Concentration (*µ*mol/L) | 0.64 | 2.26 | 9.92 |
|  | CV (%) | 4.60 | 6.32 | 4.40 |

TMAO = trimethylamine N-oxide.

**Supplementary Figure 1** Geographical locations of study sites.

**
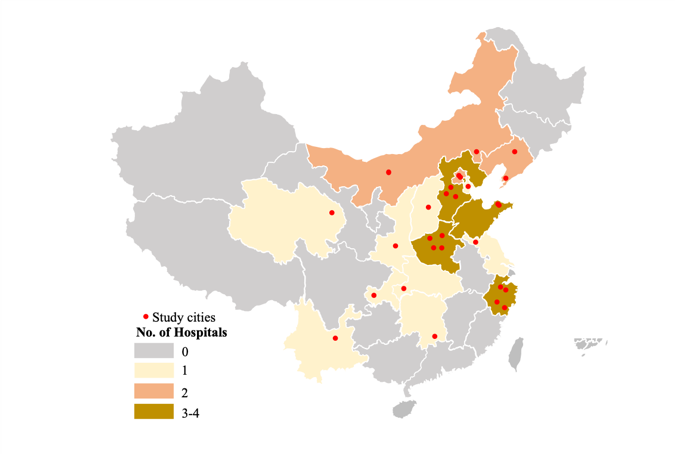
**

**Supplementary Table 1** Plasma TMAO levels between stroke subtypes

|  | **TOAST classification** | | | | |  |
| --- | --- | --- | --- | --- | --- | --- |
|  | **LAA** | **CE** | **SVO** | **OD** | **UD** | **P value** |
| **TMAO, *µ*mol/L** | 1.7 (1.2, 2.6) | 2.1 (1.4, 2.8) | 1.8 (1.2, 2.4) | 1.7 (1.0, 2.8) | 1.7 (1.2, 2.5) | 0.37 |

Data are presented as median (interquartile range)

TOAST = Trial of Org 10172 in acute stroke treatment; LAA = large-artery atherosclerosis; CE = cardio-embolism; SVO = small-vessel occlusion; OD = other determined etiology; UD = undetermined etiology

**Supplementary Table 2** Demographics and baseline characteristics of patients divided into four groups based on the severity of WMH according to the Fazekas scale

|  | **Total WMH burden** | | | |  |
| --- | --- | --- | --- | --- | --- |
| **Parameters** | **score=0**  **（n=67）** | **score=1–2**  **（n=570）** | **score=3–4**  **（n=262）** | **score=5–6**  **（n=199）** | **P value** |
| **Demographics** |  |  |  |  |  |
| **Age，yrs** | 52 (45, 57) | 59 (53, 66) | 65 (58, 71) | 68 (61, 75) | <0.0001 |
| **Male** | 50 (74.6) | 408 (71.6) | 188 (71.8) | 128 (64.3) | 0.19 |
| **Vascular risk factors** |  |  |  |  |  |
| **Current smoker** | 27 (40.3) | 208 (36.5) | 88 (33.6) | 55 (27.6) | 0.10 |
| **Hypertension** | 25 (37.3) | 340 (59.7) | 174 (66.4) | 165 (82.9) | <0.0001 |
| **Diabetes mellitus** | 10 (14.9) | 137 (24.0) | 60 (22.9) | 47 (23.6) | 0.42 |
| **Prior Stroke/TIA** | 9 (13.4) | 116 (20.4) | 79 (30.2) | 80 (40.2) | <0.0001 |
| **BMI, kg/m^2^** | 25.1 (23.0, 27.4) | 24.6 (22.9, 26.7) | 24.5 (22.5, 26.5) | 25.1 (23.3, 27.1) | 0.19 |
| **Systolic BP, mmHg** | 141 (131, 153) | 148 (132, 163) | 149 (135, 164) | 150 (135, 168) | 0.01 |
| **Diastolic BP, mmHg** | 88 (78, 93) | 88 (78, 95) | 87 (79, 95) | 88 (80, 97) | 0.61 |
| **Medical use history** |  |  |  |  |  |
| **Anti-platelet agent** | 9 (13.4) | 83 (14.6) | 49 (18.7) | 54 (27.1) | 0.0007 |
| **Lipid-lowering agent** | 7 (10.5) | 55 (9.7) | 33 (12.6) | 30 (15.1) | 0.18 |
| **Anti-hypertensive agent** | 18 (26.9) | 228 (40.0) | 123 (47.0) | 129 (64.8) | <0.0001 |
| **Laboratory tests** |  |  |  |  |  |
| **LDL-C, mmol/L** | 2.2 (1.4, 2.9) | 2.2 (1.6, 2.9) | 2.2 (1.6, 2.9) | 2.2 (1.7, 3.0) | 0.61 |
| **HDL-C, mmol/L** | 0.9 (0.7, 1.1) | 0.94 (0.8, 1.1) | 0.9 (0.8, 1.1) | 1.0 (0.8, 1.1) | 0.09 |
| **eGFR,**  **mL/min/1.73 m^2^** | 104.2 (97.7, 109.9) | 97.01 (88.0, 104.6) | 92.0 (81.9, 98.9) | 87.7 (74.6, 96.8) | <0.0001 |
| **hs-CRP, mg/L** | 1.4 (0.9, 3.3) | 1.3 (0.7, 3.3) | 1.5 (0.7, 4.4) | 2.1 (0.8, 6.1) | 0.01 |
| **Hcy, *µ*mol/L** | 14.4 (11.8, 20.0) | 16.4 (13.2, 22.6) | 17.0 (13.6, 21.3) | 19.4 (14.4, 27.5) | <0.0001 |
| **TMAO, *µ*mol/L** | 1.3 (1.0, 1.8) | 1.6 (1.1, 2.3) | 1.81 (1.26, 2.61) | 2.1 (1.4, 2.9) | <0.0001 |
| **Choline, *µ*mol/L** | 12.1 (10.4, 14.6) | 13.3 (11.2, 15.7) | 14.15 (11.8, 17.2) | 14.2 (11.9, 17.1) | <0.0001 |

Data are presented as median (interquartile range) or number (%).

WMH were scored by the Fazekas scale and the definition of "low", "intermediate", and "high" were between 1-2, 3-4 and 5-6, accordingly.

WMH = white matter hyperintensity; BMI = body mass index; BP = blood pressure; TIA = transient ischemic attack; eGFR = estimated glomerular filtration rate; LDL-C = low-density lipoprotein cholesterol; HDL-C = high-density lipoprotein cholesterol; hs-CRP = high sensitive-C-reactive protein; Hcy = homocysteine; TMAO = trimethylamine N-oxide.

**Supplementary Table 3** Comparison of demographics and baseline characteristics of patients divided into three groups based on the number of lacunes or CMBs

|  | **Lacunes** | | | | **CMBs** | | | |
| --- | --- | --- | --- | --- | --- | --- | --- | --- |
| **Parameters** | **0**  **（n=506）** | **1–2**  **（n=304）** | **>2**  **（n=288）** | **P value** | **0**  **（n=749）** | **1–2**  **（n=181）** | **>2**  **（n=168）** | **P value** |
| **Demographics** |  |  |  |  |  |  |  |  |
| **Age，yrs** | 61 (52, 68) | 63 (55, 70) | 64 (56, 70) | <0.0001 | 61 (53, 68) | 63 (56, 70) | 65 (58, 72) | <0.0001 |
| **Male** | 339 (67.0) | 221 (72.7) | 214 (74.3) | 0.06 | 526 (70.2) | 134 (74.0) | 114 (67.9) | 0.43 |
| **Vascular risk factors** | | | | | | | | |
| **BMI, kg/m^2^** | 24.5  (22.6, 26.6) | 24.8  (23.1, 27.0) | 24.9  (22.9, 26.8) | 0.04 | 24.6  (22.7, 26.6) | 24.77  (22.9, 27.0) | 25.2  (23.4, 27.0) | 0.10 |
| **Systolic BP, mmHg** | 148  (130, 161) | 148  (135, 166) | 150  (133, 165) | 0.08 | 147  (131, 162) | 150  (135, 167) | 151  (138, 168) | 0.004 |
| **Diastolic BP, mmHg** | 87 (78, 95) | 88 (80, 95) | 88 (80, 98) | 0.10 | 86 (78, 94) | 90 (80, 99) | 90 (80, 98) | 0.0004 |
| **Current smoker** | 154 (30.4) | 114 (37.5) | 110 (38.2) | 0.04 | 261 (34.9) | 60 (33.2) | 57 (33.9) | 0.90 |
| **Hypertension** | 278 (54.9) | 212 (69.7) | 214 (74.3) | <0.0001 | 424 (56.6) | 141 (77.9) | 139 (82.7) | <0.0001 |
| **Diabetes mellitus** | 118 (23.3) | 79 (26.0) | 57 (19.8) | 0.20 | 195 (26.0) | 32 (17.7) | 27 (16.1) | 0.004 |
| **Prior Stroke/TIA** | 76 (15.0) | 96 (31.6) | 112 (38.9) | <0.0001 | 165 (22.0) | 44 (24.3) | 75 (44.6) | <0.0001 |
| **Medical use history** | | | | | | | | |
| **Anti-platelet agent** | 50 (9.88) | 70 (23.0) | 75 (26.0) | <0.0001 | 108 (14.4) | 42 (23.2) | 45 (26.8) | <0.0001 |
| **Lipid-lowering agent** | 38.0 (7.5) | 46 (15.1) | 41 (14.2) | 0.0009 | 68 (9.1) | 28 (15.5) | 29 (17.3) | 0.002 |
| **Anti-hypertensive agent** | 183 (36.2) | 153 (50.3) | 162 (56.3) | <0.0001 | 284 (37.9) | 103 (56.9) | 111 (66.1) | <0.0001 |
| **Laboratory tests** | | | | | | | | |
| **LDL-C, mmol/L** | 2.2  (1.6, 2.9) | 2.3  (1.7, 3.1) | 2.2  (1.6, 2.9) | 0.17 | 2.3  (1.6, 3.0) | 2.3  (1.7, 3.0) | 2.1  (1.6, 2.8) | 0.16 |
| **HDL-C, mmol/L** | 1.0  (0.8, 1.1) | 0.9  (0.8, 1.1) | 0.9  (0.8, 1.1) | 0.60 | 0.9  (0.8, 1.1) | 0.9  (0.8, 1.2) | 1.0  (0.8, 1.1) | 0.84 |
| **eGFR,**  **mL/min1.73 m^2^** | 97.7  (88.2, 105.0) | 93.0  (80.8, 101.6) | 91.1  (80.4, 99.4) | <0.0001 | 96.1  (86.2, 104.2) | 91.8  (77.3, 100.5) | 91.2  (81.3, 98.5) | <0.0001 |
| **Hs-CRP, mg/L** | 1.4  (0.7, 3.8) | 1.6  (0.8, 3.9) | 1.4  (0.7, 3.5) | 0.50 | 1.3  (0.7, 3.9) | 1.8  (0.7, 3.7) | 1.7  (0.7, 3.6) | 0.79 |
| **Hcy, *µ*mol/L** | 15.8  (12.8, 20.0) | 16.8  (13.4, 23.3) | 19.8  (14.6, 29.0) | <0.0001 | 16.5  (13.1, 21.8) | 18.8  (14.4, 25.5) | 17..0  (13.9, 24.0) | 0.003 |
| **TMAO, *µ*mol/L** | 1.7  (1.1, 2.3) | 1.8  (1.2, 2.6) | 1.8  (1.2, 2.8) | 0.02 | 1.7  (1.2, 2.4) | 1.9  (1.3, 2.7) | 1.7  (1.2, 2.6) | 0.08 |
| **Choline, *µ*mol/L** | 13.2 (10.9, 15.9) | 13.4  (11.2, 16.2) | 14.2  (11.9, 16.9) | 0.02 | 13.3  (11.2, 15.9) | 13.9  (11.5, 16.7) | 14.1  (12.0, 17.1) | 0.09 |

Data are presented as median (interquartile range) or number (%).

CMBs = cerebral microbleeds; BMI = body mass index; BP = blood pressure; TIA = transient ischemic attack; eGFR = estimated glomerular filtration rate; LDL-C = low-density lipoprotein cholesterol; HDL-C = high-density lipoprotein cholesterol; hs-CRP = high sensitive-C-reactive protein; Hcy = homocysteine; TMAO = trimethylamine N-oxide.

**Supplementary Table 4** Relationships between plasma TMAO and choline concentrations and risk of having greater number of lacunes

|  | **Lacunes** | |
| --- | --- | --- |
|  | **Unadjusted^a^**  **OR (95% CI)** | **Adjusted^b^**  **OR (95% CI)** |
| **TMAO (range, *µ*mol/L)** |  |  |
| **1st quartile (<1.2)** | 1.0 (ref) | 1.0 (ref) |
| **2nd quartile (1.2, 1.7)** | 1.1 (0.8, 1.5) | 0.9 (0.7, 1.3) |
| **3rd quartile (1.7, 2.5)** | 0.9 (0.7, 1.3) | 0.7 (0.5, 1.1) |
| **4th quartile (>2.5)** | 1.7 (1.2, 2.3) | 1.2 (0.9, 1.8) |
| **Choline (range, *µ*mol/L)** |  |  |
| **1st quartile (<11.4)** | 1.0 (ref) | 1.0 (ref) |
| **2nd quartile (11.4, 13.5)** | 1.5 (1.1, 2.0) | 1.4 (1.0, 2.0) |
| **3rd quartile (13.5, 16.2)** | 1.6 (1.1, 2.2) | 1.5 (1.0, 2.1) |
| **4th quartile (>16.2)** | 1.6 (1.1, 2.1) | 1.3 (0.9, 1.8) |

^a^Ordinal logistic regression. ^b^Model adjusted for age, sex, hypertension, diabetes mellitus, prior stroke or transient ischemic attack, history of anti-platelet, lipid-lowering, or anti-hypertensive agents, BMI (body mass index), systolic blood pressure, low-density lipoprotein cholesterol, estimated glomerular filtration rate, homocysteine, and high sensitive-C-reactive protein.

TMAO = trimethylamine N-oxide; OR = odds ratio; CI = confidence interval.

**Supplementary Table 5** Relationships between plasma TMAO and choline concentrations and risk of having greater number of CMBs

|  | **CMBs** | |
| --- | --- | --- |
|  | **Unadjusted^a^**  **OR (95% CI)** | **Adjusted^b^**  **OR (95% CI)** |
| **TMAO (range, *µ*mol/L)** |  |  |
| **1st quartile (<1.2)** | 1.0 (ref) | 1.0 (ref) |
| **2nd quartile (1.2, 1.7)** | 1.2 (0.8, 1.7) | 1.1 (0.7, 1.6) |
| **3rd quartile (1.7, 2.5)** | 1.1 (0.7, 1.5) | 1.0 (0.6, 1.4) |
| **4th quartile (>2.5)** | 1.3 (0.9, 1.9) | 0.9 (0.6, 1.4) |
| **Choline (range, *µ*mol/L)** |  |  |
| **1st quartile (<11.4)** | 1.0 (ref) | 1.0 (ref) |
| **2nd quartile (11.4, 13.5)** | 1.1 (0.8, 1.6) | 0.9 (0.6, 1.4) |
| **3rd quartile (13.5, 16.2)** | 1.2 (0.8, 1.7) | 1.1 (0.7, 1.7) |
| **4th quartile (>16.2)** | 1.5 (1.0, 2.1) | 1.2 (0.8, 1.8) |

^a^Ordinal logistic regression. ^b^Model adjusted for age, sex, hypertension, diabetes mellitus, prior stroke or transient ischemic attack, history of anti-platelet, lipid-lowering, or anti-hypertensive agents, BMI (body mass index), systolic blood pressure, low-density lipoprotein cholesterol, estimated glomerular filtration rate, homocysteine, and high-sensitive-C-reactive protein.

TMAO = trimethylamine N-oxide; CMB = cerebral microbleed; OR = odds ratio; CI = confidence interval.
